# Evidence for maternal control of seed size in maize from phenotypic and transcriptional analysis

**DOI:** 10.1093/jxb/erw006

**Published:** 2016-01-29

**Authors:** Xia Zhang, Candice N. Hirsch, Rajandeep S. Sekhon, Natalia de Leon, Shawn M. Kaeppler

**Affiliations:** ^1^Department of Agronomy, University of Wisconsin-Madison, 1575 Linden Drive, Madison, WI 53706, USA; ^2^Department of Agronomy and Plant Genetics, University of Minnesota, St. Paul, MN 55108, USA; ^3^Department of Genetics and Biochemistry, Clemson University, Clemson, SC 29634, USA; ^4^DOE Great Lakes Bioenergy Research Center, University of Wisconsin-Madison, 1575 Linden Drive, Madison, WI 53706, USA

**Keywords:** Endosperm, gene expression, maize, maternal effect, seed development, seed size.

## Abstract

Seed size is an important component of grain yield and a key determinant trait for crop domestication. The Krug Yellow Dent long-term selection experiment for large and small seed provides a valuable resource to dissect genetic and phenotypic changes affecting seed size within a common genetic background. In this study, inbred lines derived from Krug Large Seed (KLS) and Krug Small Seed (KSS) populations and reciprocal F_1_ crosses were used to investigate developmental and molecular mechanisms governing seed size. Seed morphological characteristics showed striking differences between KLS and KSS inbred lines, and the reciprocal cross experiment revealed a strong maternal influence on both seed weight and seed size. Quantification of endosperm area, starchy endosperm cell size, and kernel dry mass accumulation indicated a positive correlation between seed size, endosperm cell number, and grain filling rate, and patterns of grain filling in reciprocal crosses mirrored that of the maternal parent. Consistent with the maternal contribution to seed weight, transcriptome profiling of reciprocal F_1_ hybrids showed substantial similarities to the maternal parent. A set of differentially expressed genes between KLS and KSS inbreds were found, which fell into a broad number of functional categories including DNA methylation, nucleosome assembly, and heat stress response. In addition, gene co-expression network analysis of parental inbreds and reciprocal F_1_ hybrids identified co-expression modules enriched in ovule development and DNA methylation, implicating these two processes in seed size determination. These results expand our understanding of seed size regulation and help to uncover the developmental and molecular basis underlying maternal control of seed size in maize.

## Highlight

An integrative study combining phenotypic and transcriptional analysis reveals extensive maternal control of seed size in maize, contributing to a better understanding of the developmental and molecular basis of seed size regulation.

## Introduction

Seed size is a key determinant for evolutionary fitness and is also a crucial agronomic trait selected during crop domestication (Doebley *et al.*, 2006). Seeds produced by cereal crops are a major source of staple food, livestock feed, and biofuel (Makkar, 2012). Seed size has been proposed to be a key contributor to grain yield in crop plants (Kesavan *et al.*, 2013; Zhang *et al.*, 2014). In maize (*Zea mays* L.) breeding programs, seed size is an important breeding target because of the requirements of both end-use quality and consumer preference (Gupta *et al.*, 2006). Understanding determinants of seed size is, therefore, essential to meet increasing demand for food staples and renewable energy by the ever-growing human population.

Seeds in angiosperms consist of three genetically distinct constituents: the embryo, the endosperm, and the seed coat. Seed development begins with a double fertilization event in which one sperm nucleus fuses with the haploid egg and produces the diploid embryo, and the other sperm nucleus fertilizes with the diploid central cell to give rise to the triploid endosperm, which is responsible for the transfer of nutrients to the embryo. The embryo and endosperm develop within the maternal tissues of the ovule, and the integuments of the ovule ultimately give rise to the coat of the mature seed (Chaudhury *et al.*, 2001). Seed size is co-ordinately determined by the growth of the triploid endosperm, the diploid embryo, and resources and developmental cues provided by the maternal plant (Sundaresan, 2005).

In monocots, the endosperm constitutes the majority of the mature seed, and endosperm size has been found to play a major role in determining seed size (Berger *et al.*, 2006). Seed size frequently depends on the development and the amount/size of the endosperm, and a relationship between endosperm cell number and seed size has been observed (Chojkecki *et al.*, 1986). Final seed size and weight are influenced by a number of cellular processes, as well as genetic and environmental factors. Genetic factors that regulate seed size zygotically or maternally have been identified in Arabidopsis as well as in crop plants (reviewed by Kesavan *et al.*, 2013; Li and Li, 2015). Epigenetic marks in the genome are also important factors affecting seed size, and genomic imprinting, primarily conveyed by DNA methylation, has been proposed as another important phenomenon affecting seed size (Gehring *et al.*, 2004; Jiang and Köhler, 2012; Fatihi *et al.*, 2013).

The maternal parent contributes to the offspring seed phenotype in multiple ways including providing photosynthate and nutrients to support development, co-ordinating developmental timing, as well as imprinting of maternal gametes. The maternal plant affects seed size via (i) the seed coat, which comprises the maternal genotype and imposes mechanical constraints on seed development; (ii) maternal provisioning during seed development; (iii) maternal determination of progeny plasticity in response to developmental signals and environmental cues; and (iv) the effect of the triploid endosperm where gene imprinting occurs most often (Roach and Wulff, 1987; Platenkamp and Shaw, 1993; Adamski *et al.*, 2009; Donohue, 2009; Fang *et al.*, 2012). Maternal nutrient allocation is important for seed development. Highly specialized maize cells in the basal endosperm transfer cell layer facilitate the transport of maternal solutes and nutrients at the interface between maternal tissues and the endosperm (Gómez *et al.*, 2002). *Zea mays MYB-related protein-1* (*ZmMRP-1*) is a gene encoding a known endosperm transfer cell-specific transcriptional activator, which is involved in the expression of basal endosperm transfer layer (BETL)-specific genes including *BETL-1*, *Basal layer-type antifungal protein 2* (*BAP2*), and *Maternally expressed gene 1* (*Meg1*), and plays a central role in the regulatory pathways controlling transfer cell differentiation and associated maternal nutrition allocation (Gómez *et al.*, 2009; Costa *et al.*, 2012; Xiong *et al.*, 2014).

Although a few known maize genes acting in endosperm and maternal tissues to affect seed development have been identified such as *miniature 1* (*mn1*) and *shrunken-2* (*sh2*) (Miller and Chourey, 1992; Hannah *et al.*, 2012), relatively little is known about the maternal genetic factors and molecular mechanisms that regulate seed size in maize. Previous studies have documented developmental timing contributions and possible genetic regions under selection in the Krug Yellow Dent long-term selection experiment for small and large seed size (Hirsch *et al.*, 2014; Sekhon *et al.*, 2014). In this study, we used inbred lines derived from the Krug Large Seed (KLS) and Krug Small Seed (KSS) populations and their reciprocal F_1_ hybrids to explore maternal determinants underlying seed size regulation, and presented evidence that the maternal parent plays an important role in determining seed size via seed morphological, cytological, and transcriptional analyses.

## Materials and methods

### Plant materials, growth conditions, and sampling details

Thirty cycles of divergent mass selection for seed size were performed in the open pollinated population Krug Yellow Dent to generate KLS30 (selected for large seed size; PI 636488) and KSS30 (selected for small seed size; PI 636489) (Odhiambo and Compton, 1987; Russell, 2006). Inbred lines were subsequently developed from the populations by self-pollination for at least seven generations without any selection for seed characteristics. This study included two KLS30-derived inbred lines (KLS_S6_1-1 and KLS_S5_2-1-1, abbreviated to L1L1 and L3L3, respectively) and two KSS30-derived inbred lines (KSS_S4_4-1-1 and KSS_S4_3-2-1, abbreviated to S1S1 and S3S3, respectively). Experiments were planted at the University of Wisconsin, West Madison Agricultural Research Station during the 2013 and 2014 growing season under the same field growing conditions previously described (Sekhon *et al.*, 2014). Briefly, genotypes were arranged in three-row plots with three replications and hand-planted in 2.9 m long rows with row and plant spacing of 0.76 m and 0.24 m, respectively. Eight F_1_ reciprocal hybrids (L1S1, L1S3, L3S1, L3S3, S1L1, S3L1, S1L3, and S3L3) were generated from these four parental inbred lines by manual pollination. These hybrids are coded such that the character on the left signifies the maternal parent and the character on the right signifies the paternal parent. Ample pollen was used for each pollination to ensure well-filled ears for consistent kernel phenotyping. Kernels from the center of three different primary ears per plot were either dried for seed weight, fixed in ethanol for imaging, or stored at –80 °C for transcriptional analysis.

### Seed weight and seed size measurement

The bulk mature seeds from each plot were used for calculating 100-seed weight measured in grams. To generate the grain filling rate, 10 kernels per ear were sampled at each time point [11, 14, 17, 20, 25, and 28 days after pollination (DAP)] with six replications. Kernel dry weight was determined after drying samples at 65 °C for 1 week. To obtain the seed size distribution, images were captured using an Epson Perfection V700 Photo desktop scanner and VueScan scanning software without image enhancement and saved as TIFF (tagged image file format) files. One hundred mature dry seeds were spread uniformly with the embryo facing the glass platen and scanned at a resolution of 1200 dpi. Using MatLab image analysis software, the major (depth) and minor (width) axes and total area of the kernels was quantified.

### Microscopic examination of endosperm area and endosperm cell size

Kernels freshly isolated from the middle of developing ears at 17 DAP were fixed in 70% ethanol (v/v) and stored in 4 °C. Kernels were first rinsed with distilled water twice and trimmed on both sides to form a 2–3mm thick longitudinal median section containing the embryo. The slices were imaged with a Zeiss AxioZoom fluorescence stereo microscope to obtain the endosperm area. These slices were also stained with 0.1% (w/v) berberine sulfate for 5–15min depending on the slice thickness to visualize the cell wall on a Zeiss LSM 510 META confocal microscope. A rectangular region of interest of the approximate location covering most of the endosperm area at 17 DAP was selected and extracted from at least 10 kernels of each of the KLS and KSS inbred lines. Endosperm size and endosperm cell size were measured using ImageJ software (http://rsbweb.nih.gov/ij/). Confocal imaging was performed at the Newcomb Imaging Center, Department of Botany, University of Wisconsin-Madison.

### RNA sequencing

Total RNA of whole developing kernels from the four parental lines and eight reciprocal F_1_ hybrids at 14 DAP and 17 DAP was extracted using TRIZOL reagent (Ambion, http://www.lifetechnologies.com), checked for quality, and further purified using an RNeasy MinElute Cleanup kit (Qiagen, http://www.qiagen.com) following the manufacturer’s instructions. Isolation of mRNA, cDNA synthesis, and construction and sequencing of RNA sequencing (RNA-Seq) libraries were performed at the University of Wisconsin Biotechnology Center (Madison, WI) using the Illumina TruSeq RNA sample preparation kit v2 protocol (Illumina, http://www.illumina.com). Sixteen samples were pooled per lane and sequenced using an Illumina HiSeq 2500 to generate 151 nucleotide paired-end sequence reads. Raw sequence reads are available through the National Center for Biotechnology Information Sequence Read Archive (BioProject PRJNA287557). Quality of the raw sequences was checked using the FastQC program (http://www.bioinformatics.babraham.ac.uk/projects/fastqc/) and reads were trimmed to 100 nucleotides to remove low quality bases with the fastx_trimmer program within the FASTX toolkit (http://hannonlab.cshl.edu/fastx_toolkit/index.html). Reads were mapped to the B73 version 2 reference sequence (Schnable *et al.*, 2009) using Bowtie version 0.12.7 (Langmead *et al.*, 2009) and TopHat version 1.2.0 (Trapnell *et al.*, 2009), setting a minimum intron length of five nucleotides and a maximum intron length of 60 000 nucleotides. Fragments per kilobase of exon model per million fragments mapped (FPKM) values were estimated by Cufflinks version 0.9.3 (Trapnell *et al.*, 2010) using the version 5b annotation and providing genome assembly, and requiring a minimum intron size of five nucleotides.

### Identification of differentially expressed genes, gene annotation, and functional enrichment

Hierarchical clustering was conducted on log_2_-transformed FPKM values of expressed genes with FPKM values >1 in all samples using the hclust command in R. Differentially expressed genes (DEGs) were identified by pairwise comparisons using edgeR (Robinson *et al.*, 2010) and read counts calculated with the coverageBed program within BEDTools version 2.17.0 (Quinlan and Hall, 2010). Only genes with read counts >1 were used for differential expression analysis, and the significance of differences in expressed genes was judged on two criteria: FDR (*P*-value after adjusting for false discovery rate) ≤0.05 and |log_2_ fold change| ≥1. A heatmap with dendrograms was produced with the pheatmap R package (Kolde, 2013). Annotation of transcriptional factor family members was based on information from GrassTFDB of GRASSIUS (Gray *et al.*, 2009; Yilmaz *et al.*, 2009). Gene Ontology (GO) enrichment analyses of the DEGs and weighted gene co-expression network analysis (WGCNA)-generated co-expression modules were performed with the goseq package in R using the Wallenius approximation method (Young *et al.*, 2010). GO term annotations for maize genes were obtained from Gramene (ftp://ftp.gramene.org/pub/gramene/CURRENT_RELEASE/data/ontology/go/). All calculations and plotting were performed in R.

### Identification of gene co-expression modules

Gene co-expression module assignments were determined using the WGCNA protocol (Zhang and Horvath, 2005; Langfelder and Horvath, 2008) based on FPKM data. Genes with FPKM <1 for all samples were filtered out, and a coefficient of variation cut-off of 0.25 was used to filter genes with low variation among samples. The Dynamic Tree Cut algorithm with a minimum module size of 50 genes was used to cut the hierarchal clustering. The soft threshold power beta was set to nine. Significant module–trait associations were identified by correlating module eigengenes with seed weight, and the modules with *P*-values <0.001 were selected for GO enrichment analysis with the goseq package in R using the Wallenius approximation method (Young *et al.*, 2010).

## Results

### Maternal parent has a significant effect on seed weight and seed size

**Fig. 1.**
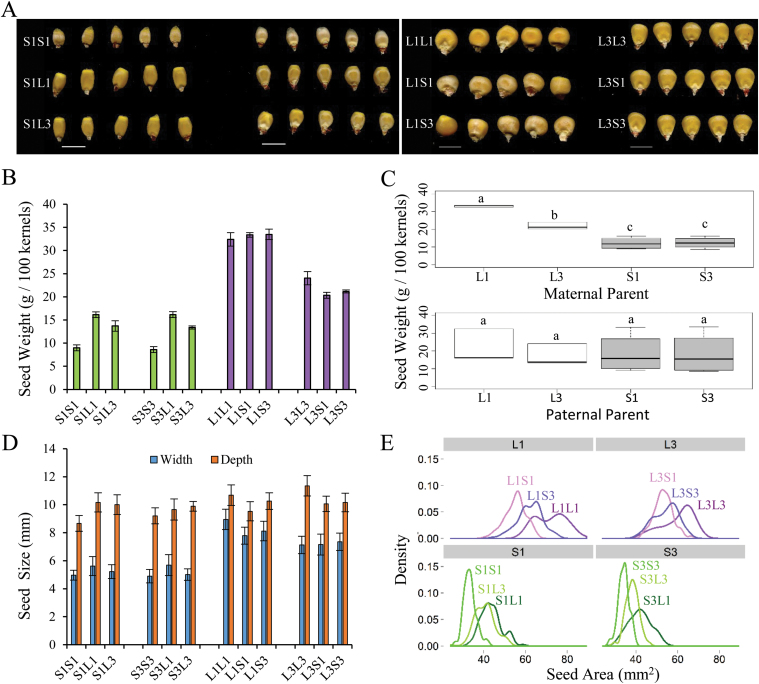
Analysis of seed weight and seed size in KLS30- and KSS30-derived inbred lines and their reciprocal F_1_ hybrids. (A) Mature seeds of inbreds and F_1_ reciprocal hybrids. S1S1, S3S3, L1L1, and L3L3 are inbred lines derived from KSS30 and KLS30, respectively. F_1_ reciprocal hybrids (e.g. S1L1) are designated with the maternal parent on the left and the paternal parent on the right. Scale bar=10mm. (B) Seed weight per 100 seeds (mean ±SD, *n*=3). (C) Parental effects on seed weights. Seeds were divided into four groups based on the common maternal parent (upper panel) and paternal parent (lower panel). The letters indicate significantly different statistical groups according to ANOVA (Tukey tests, *P*<0.05). (D) Average seed width and seed depth quantified by image analysis from 100 seeds (error bars=SD of 100 seeds). (E) Density plot of the distribution of seed area between maternal groups. Measurements were from image analysis of 100 seeds. L1, L3, S1, and S3 refer to the maternal parents shared in the group.

Significant variation for seed size among the KLS30 and KSS30 populations has previously been shown (Odhiambo and Compton, 1987; Russell, 2006; Sekhon *et al.*, 2014). To understand the maternal contribution to this variation, two KLS inbred lines (named L1L1 and L3L3) and two KSS inbred lines (named S1S1 and S3S3), derived from KLS30 and KSS30, respectively, and their reciprocal F_1_ crosses were developed (Fig. 1A). The dry weight of the mature seeds of parental inbred lines showed that KLS inbreds had 267–377% of the seed weight of KSS inbreds. A strong maternal influence on seed weight was remarkable as hybrid seeds produced with KLS inbreds as the mother plants, irrespective of the genotype of the pollen donor, were consistently heavier than those produced by maternal KSS plants (Fig. 1B). The significant maternal effects on seed weight were further revealed by plotting samples based on the maternal or paternal parents they had in common (L1, L3, S1, and S3) (Fig. 1C). When evaluated among maternal parent groups (Fig. 1C, upper panel), seed weights of maternal group L (L1 and L3) and maternal group S (S1 and S3) were significantly different (Tukey test, *P*<0.05) and there was no overlap in the spread between group L and group S. However, for the paternal groups, the spread overlapped across all four group medians (Fig. 1C, lower panel), and no significant differences were observed for paternal effects (Tukey test, *P*>0.05).

We further quantified seed size by image analysis. Consistent with seed weight, kernel width and depth of KSS inbreds were significantly smaller than those of KLS inbreds (Student’s *t*-test, *P*<0.05). Comparisons between group samples that shared either the same KSS maternal parent or the same KLS maternal parent only showed a significant difference in kernel width (Fig. 1D). Frequency histograms of kernel area corroborated the maternal effect, showing the clear skew trend that discriminated the KLS and KSS maternal groups (Fig. 1E). Together, the seed morphology analysis revealed a striking difference in seed weight and seed size between KLS and KSS parental inbreds as well as the significant contribution of the maternal parent to seed weight and seed size in reciprocal hybrids.

### KLS inbred lines have larger endosperms and smaller cells than KSS inbred lines, and hybrids mirror the developmental rate of the maternal parent

**Fig. 2.**
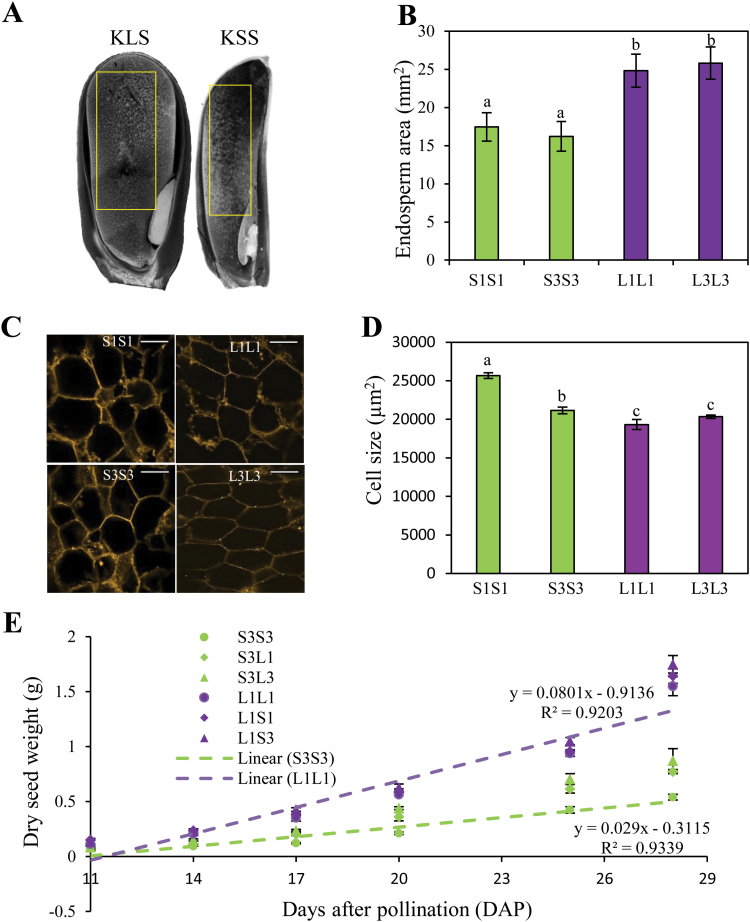
Comparison of endosperm area and cell size of KLS and KSS inbreds as well as grain filling rate in inbreds and hybrids. (A) Representative endosperms of KLS and KSS inbred lines at 17 days after pollination (DAP). Endosperm zones for measuring cell size are highlighted by the yellow rectangles. (B) Endosperm area of KLS and KSS inbreds at 17 DAP. Measurements are taken for each genotype on 33–54 developing kernels from three ears (means ±SD). Means with different letters are significantly different from each other (Tukey’s multiple pairwise comparisons test, *P*<0.05). (C) Endosperm cells of KLS and KSS inbred lines. Kernel sections dissected as described in the Materials and methods were stained with berberine sulfate and observed with a confocal microscope. Representative images are shown at ×20 magnification. Scale bars=100 µm. (D) Cell size in the endosperms of KLS and KSS inbreds at 17 DAP. Each data point represents 300–500 measurements (means ±SE). Different letters indicate significant differences according to ANOVA (Tukey’s test, *P*<0.05). (E) Grain filling rate in KLS and KSS inbreds and the corresponding hybrids. For clear demonstration, shown here is a time course of kernel dry weight collected from one KLS inbred (L1L1) and one KSS inbred (S3S3) and their crosses with two other inbred lines (S3L1/S3L3 and L1S1/L1S3). Values are the mean ±SD for *n*=6 replicates from three ears per date, 10 kernels per replicate. Trend line equations are shown for the inbred lines. S1S1, S3S3, L1L1, and L3L3 are inbred lines derived from KSS30 and KLS30, respectively. F_1_ reciprocal hybrids (e.g. S3L1) are designated with the maternal parent on the left and the paternal parent on the right.

The endosperm in cereals is the main nutrient sink where storage materials are deposited during grain filling. To explore if variation in seed weight and seed size was associated with changes in endosperm characteristics, we compared endosperm area and starchy endosperm cell size between KSS and KLS inbreds. The median longitudinal sections that contained the embryo of kernels collected at 17 DAP were selected for measuring endosperm area, and a large rectangular region covering the majority of the starchy endosperm was used for measuring cell size (Fig. 2A). Overall, KLS inbreds had a 30–38% larger endosperm area compared with KSS inbreds (Fig. 2B). Surprisingly, cell area was smaller in KLS inbreds (Tukey test, *P*<0.05). L1L1 endosperm had smaller cell area than S1S1 and S3S3 by 32% and 9%, respectively, and this reduction for L3L3 was 26% and 4% in comparison with S1S1 and S3S3, respectively (Fig. 2C, D). The larger endosperms and smaller cell size of KLS inbreds compared with KSS inbreds indicates that KLS inbreds have a higher number of endosperm cells, and that the difference in seed size between KLS and KSS inbreds would be mainly explained by cell number rather than cell size.

Grain filling is an important agronomic trait in cereals, where a number of cell layers in the endosperm play a critical role. We systematically evaluated the performance of parental inbreds and derived hybrids for the grain filling rate by measuring dry mass accumulation throughout kernel development beginning at 11 DAP. KLS inbreds and hybrids with a KLS inbred as the maternal parent accumulated dry matter faster than KSS inbreds and hybrids with a KSS inbred as the maternal parent. Importantly, hybrids exhibited remarkable resemblance to the maternal parent in the rate of grain filling (Fig. 2E).

### Global gene expression pattern of Krug reciprocal F_1_ hybrids exhibits substantial similarities with maternal parents

**Fig. 3.**
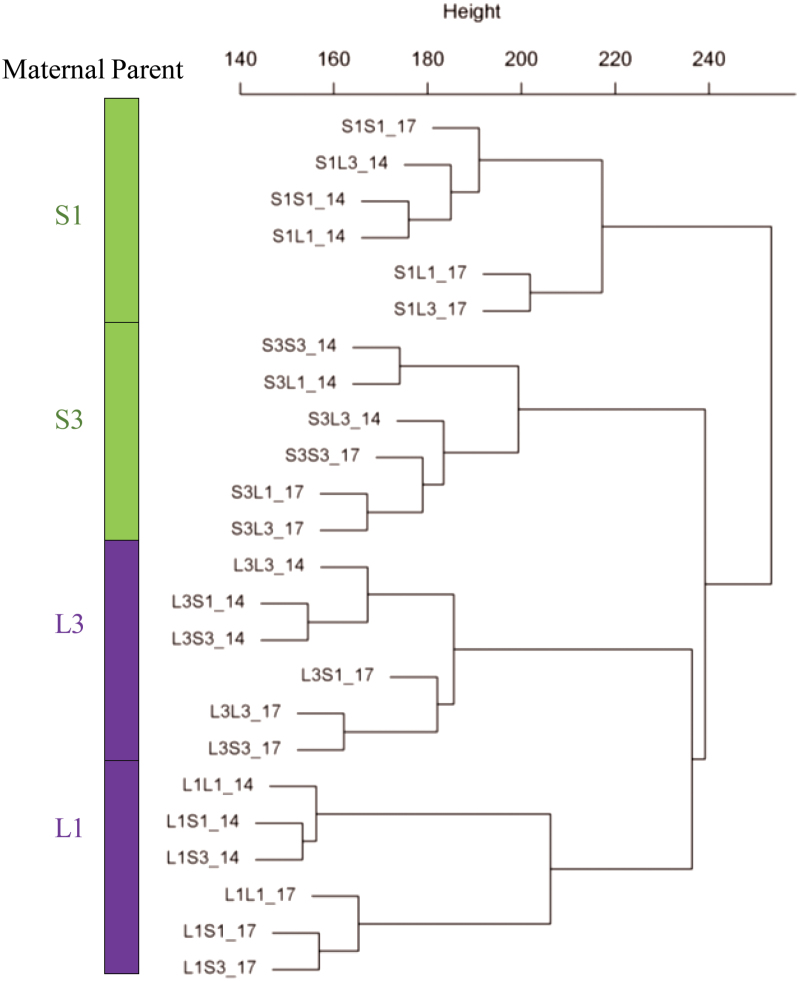
Transcriptome profile of inbred lines derived from KSS30 and KLS30 and their reciprocal hybrids at 14 and 17 days after pollination (DAP). Hierarchical clustering of log_2_ transformation of the expressed genes (fragments per kilobase of exon model per million fragments mapped >1) was used to generate the tree. _14 and _17 indicate samples collected at 14 DAP and 17 DAP, respectively. S1S1, S3S3, L1L1, and L3L3 are inbred lines derived from KSS30 and KLS30, respectively. F_1_ reciprocal hybrids (e.g. S1L1) are designated with the maternal parent on the left and the paternal parent on the right. S1, S3, L3, and L1 refer to the common maternal parent shared in the group samples.

In the context of understanding the molecular mechanisms and genetic regulation underlying seed size control, we profiled the transcriptomes of developing kernels of KSS and KLS inbred lines and their F_1_ reciprocal hybrids collected at 14 DAP and 17 DAP. We generated 11×10^6^–18×10^6^ raw reads for each sample, of which 81.5–84.7% aligned to the B73 v2 reference genome assembly (Schnable *et al.*, 2009); the unique aligned reads accounted for ~75% of the sequenced raw reads (Supplementary Table S1 at *JXB* online). The relative abundance of transcripts calculated as the FPKM was provided (Supplementary Table S2). We found that 19 909–22 443 of 39 456 maize gene models were expressed with FPKM >1 among the samples (Supplementary Table S1). Intriguingly, hierarchical clustering of the transcriptome profiles grouped the samples into four major clades corresponding to the four maternal parents, and reciprocal crosses always formed the primary cluster with their maternal parent (Fig. 3). This similarity in expression pattern between reciprocal crosses and their maternal parents provides valuable insights into the regulatory role of the maternal transcriptome in phenotypic variation for seed size and seed weight.

### Identification of differentially expressed genes between KLS and KSS inbreds

**Fig. 4.**
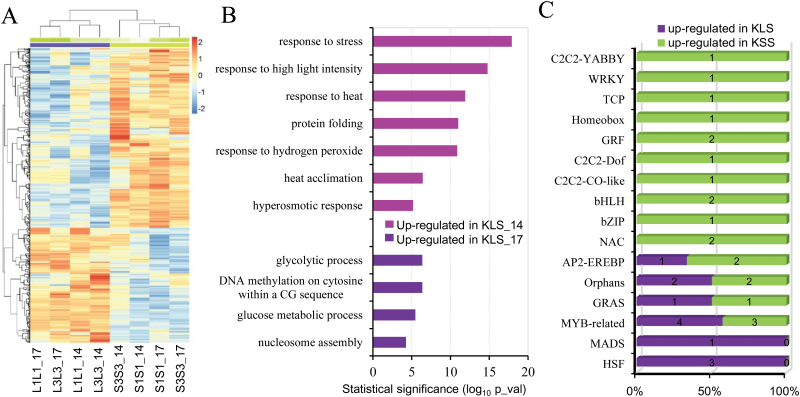
Differentially expressed genes (DEGs) between inbred lines derived from the KSS30 and KLS30 populations. (A) Heatmap of DEGs with 2-fold up- or down-regulation between KLS and KSS inbreds (KLS versus KSS) at 14 DAP and 17 DAP. (B) Significantly enriched Gene Ontology (GO) categories with the up-regulated genes in KLS inbred lines. (C) Distribution of transcription factor families among DEGs. The percentage on the *x*-axis was plotted as the number of differentially expressed transcription factors in each genotype out of all the differential transcription factors identified in the family. _14 and _17 indicate samples collected at 14 DAP and 17 DAP, respectively. S1S1, S3S3, L1L1, and L3L3 are inbred lines derived from KSS30 and KLS30, respectively.

Treating inbred lines of each genotype as replicates, we identified 402 and 691 DEGs between KLS and KSS inbreds at 14 DAP and 17 DAP, respectively. Of these, 187 and 229 showed higher transcript abundance in KLS inbreds at 14 DAP and 17 DAP, and 215 and 462 DEGs were more abundant in KSS inbreds at 14 DAP and 17 DAP, respectively (Fig. 4A; Supplementary Table S3). GO enrichment analysis was performed on these four groups of DEGs to discover over-represented functional categories (Fig. 4B; Supplementary Table S3). No significant GO terms were found enriched in DEGs up-regulated in KSS inbreds at 14 DAP, while up-regulated DEGs in KSS inbreds at 17 DAP were enriched in major metabolic processes such as ‘carbohydrate metabolic process’ and ‘ֲfatty acid metabolic process’; nutrient reservoir activity was one subcategory of molecular function showing the most marked over-representation in KSS inbreds at both 14 DAP and 17 DAP (see Supplementary Table S3). Significantly over-represented GO terms among DEGs up-regulated in KLS inbreds at 14 DAP were all related to stimulus responses (‘response to stress’, ‘response to high light intensity’, ‘response to heat’, ‘protein folding’, ‘response to hydrogen peroxide’, ‘heat acclimation’, and ‘hyperosmotic response’) (Fig. 4B), suggesting improved responses or adaptation to environmental cues. Significant functional over-representations enriched in up-regulated genes in KLS inbreds at 17 DAP included ‘glycolytic process’, ‘DNA methylation’, ‘glucose metabolic process’, and ‘nucleosome assembly’ (Fig. 4B). Genes enriched for nucleosome assembly, a biological process also involved in DNA replication and cell division, mainly included histone superfamily genes, which are crucial for packaging of DNA and cell cycle regulation (Marzluff and Duronio, 2002). Up-regulated genes in KLS at 17 DAP related to DNA methylation were linked to three genes required for maintenance of CG methylation in plants, namely VARIANT IN METHYLATION 103 (*VIM103*) and two DNA methyltransferase genes (*MET8* and *MET1*) (Feng *et al.*, 2010; Law and Jacobsen, 2010; Candaele *et al.*, 2014). DNA methylation is a key epigenetic determinant that regulates gene imprinting in plants, and imprinting has been proposed to be involved in maternal control of nutrient distribution in plant seeds (Feil and Berger, 2007; Costa *et al.*, 2012).

We also examined differential accumulation of transcription factors between KLS and KSS inbred lines. Thirty-three transcription factors representing 16 families were identified as differentially expressed in KLS and KSS inbreds (Fig. 4C; Supplementary Table S4). Of the transcription factors up-regulated in KSS inbreds, Prolamin-box binding factor1 (Pbf1) and WRINKLED1 transcription factor 2 (ZmWri1b) from the DOF family and AP2/EREBP gene family, respectively, have been documented as regulators of storage protein and seed oil (Pouvreau *et al.*, 2011; Lang *et al.*, 2014; Zhang *et al.*, 2015). Their up-regulation in KSS inbreds largely corroborated GO enrichment analysis of DEGs. In addition, transcription factors that function in response to environmental cues and nutrient uptake and transport, including three heat shock factors (ZmHSF17, ZmHSF20, and ZmHSF24) (Yilmaz *et al.*, 2009) and one MYB-related protein ZmMRP-1 (Gómez *et al.*, 2002), were up-regulated in KLS inbreds. ZmMRP-1 is known as a primary endosperm transfer cell-specific transcriptional activator that plays a central role in the regulatory pathways controlling transfer cell differentiation and associated maternal nutrition allocation (Gómez *et al.*, 2009). Together, functional characterization of DEGs between KLS and KSS inbreds indicated that different adaptive responses to environmental and developmental cues could influence their ability to provision seeds and therefore affect offspring phenotypes when serving as maternal plants.

### Gene co-expression network identifies biological processes and candidate genes important for maternal effects on seed size

**Fig. 5.**
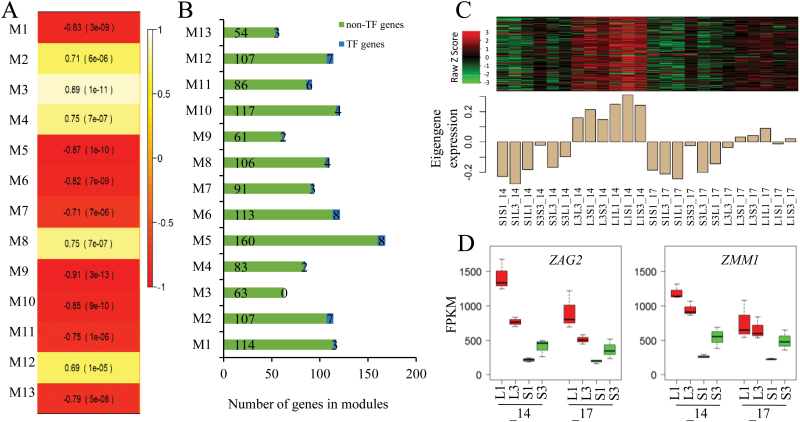
Identification of co-expression module genes associated with seed weight using the WGCNA method. (A) Module–trait relationship showing the significance of the module eigengene correlation with seed weight. Shown are correlations and *P*-values; cell color denotes correlation (white, positive correlation; red, negative correlation according to the color key). (B) Distribution of gene number including non-transcription factors and transcription factors among the modules that were significantly associated with seed weight. (C) Heatmap of M12 gene expression (upper panel) and expression levels of the corresponding eigengene across the samples in the M12 module (lower panel). The heatmap (upper panel) and barplot of eigengene expression (lower panel) have the same samples (*x*-axis). Rows of the heatmap correspond to genes, columns to samples; red in the color key denotes overexpression, green underexpression. (D) Differential expression of *ZAG2* and *ZMM1* in all samples grouped by maternal genotypes at 14 DAP (_14 in axis) and 17 DAP (_17 in axis). S1, S3, L1, and L3 signify the maternal parents that the samples have in common. _14 and _17 indicate samples collected at 14 DAP and 17 DAP, respectively. S1S1, S3S3, L1L1, and L3L3 are inbred lines derived from KSS30 and KLS30, respectively. F_1_ reciprocal hybrids (e.g. S1L1) are designated with the maternal parent on the left and the paternal parent on the right.

To identify networks of co-expressed genes, especially those that are correlated with seed size, we performed a WGCNA. After CV filtering, 6349 expressed genes (FPKM ≥1) in our transcriptome profiling fell into 52 modules, with each containing at least 50 genes (Supplementary Fig. S1; Supplementary Table S5). The identified modules were then selected on the basis of the module–trait relationship, which was calculated by correlating the module’s eigengene value to the mature seed weight. Thirteen of these modules were found strongly correlated with seed weight (correlations ranging from –0.87 to 0.89, *P*<0.001), containing between 57 (M13) and 168 (M5) genes (Fig. 5A), and including two (M4) to eight (M5 and M6) transcription factor genes (Fig. 5A, B). Among the eight modules negatively correlated with seed weight, four of them (M1, M5, M10, and M13) showed differential expression patterns between the KSS group (KSS inbreds and hybrids with a KSS inbred as the maternal parent) and the KLS group (KLS inbred lines and hybrids with a KLS inbred as the maternal parent) (Supplementary Fig. S2). The molecular functions and biological processes that were most significantly enriched in these modules negatively correlated with seed weight were protein autophosphorylation (M1), nutrient reservoir activity (M5), hexose catabolic process (M10), and trehalose biosynthetic process (M13) (Table 1). M4 and M12 were two of the five modules positively correlated with seed weight, both of which showed consistently lower expression in the KSS group compared with the KLS group at both 14 DAP and 17 DAP (Fig. 5C). Consistent with GO enrichment analysis of DEGs, M4 was significantly enriched in DNA methylation (Table 1), which included *MET1* and *MET8*. M12 contained 114 genes including seven transcription factor genes from the families ARF, bZIP, G2-like, MADS-box, and Orphans. Over-represented biological processes of M12 were related to floral organ development including maintenance of floral organ identity, carpel development, and ovule development (Table 1). This module mainly involved three MADS-box transcription factor genes: *Zea mays AGAMOUS homolog 2* (*ZAG2*), *Zea mays MADS1* (*ZMM1*), and *ZMM*2. According to the maize gene expression atlas (Sekhon *et al.*, 2011; Stelpflug *et al.*, 2015), *ZAG2* and its paralogous gene *ZMM1* are largely restricted to reproductive organs, whole seed, endosperm, and pericarp (Supplementary Fig. S3). Interestingly, *ZAG2* has been identified as a maternally expressed imprinted gene in the maize endosperm (Liu *et al.*, 2015). Together, the WGCNA analysis largely corroborated findings from standard differential gene expression analyses, and also identified possible meta-networks composed of multiple GO categories underlying the observed maternal effect on seed size.

**Table 1.** Functional module gene enrichment characterization including number of genes, correlation with seed weight, and attributes of the most significant GO terms

Network module	Modulet–trait relationship	Most significant GO term	*P*-value
M1	Negative	GO:0046777 protein autophosphorylation	5.6e-3
M5	Negative	GO:0045735 nutrient reservoir activity	1.4e-6
M10	Negative	GO:0006096 glycolytic process	2.3e-5
M13	Negative	GO:0005992 trehalose biosynthetic process	1.9e-3
M4	Positive	GO:0010424 DNA methylation on cytosine within a CG sequence	1.1e-4
M12	Positive	GO:0048481 ovule development	6.7e-6

## Discussion

Variation in seed size is common within and among plant species. Underlying this variation, and thus regulation of seed size, is a complex array of interactions involving genetic factors, developmental signals, and environmental cues. Although maternal effects in plants have long been recognized (Roach and Wulff, 1987), the mechanisms whereby maternal effects affect seed size remain largely unknown, which is especially true for maize. By using a unique genetic resource derived from the Krug Yellow Dent long-term selection experiment for seed size in maize, we identified remarkable reciprocal differences due to large maternal effects on seed weight and seed size. Integrative analysis of seed morphogenesis (endosperm size and grain filling) and transcriptome profiling further provides insights into developmental and molecular events underlying the maternal control of seed size.

Our observation that reciprocal F_1_ crosses closely mirrored the phenotype of the self-pollinated maternal parent in terms of seed weight, seed size, and seed development provides strong support for a maternal influence on seed size in maize. The endosperm in cereals serves as the primary nutrient source for embryo and seed development. The endosperm development depends on both sink capacity and assimilates supplied by sporophytic maternal tissues, thus implicating the maternal genotype in the process. The endosperm’s strength as a nutrient sink is proposed to be the function of the number of endosperm cells and/or the number of starch granules formed during grain filling (Capitanio *et al.*, 1983; Reddy and Daynard, 1983). Maternal effects on kernel mass are thought to be due to changes in the number of endosperm cells formed (Jones *et al.*, 1996). In our study, KLS inbred lines have larger endosperms but smaller cells compared with KSS inbreds (Fig. 2), indicating more endosperm cells in the large seed genotype. Thus, maternal sink constraint determined by the number of endosperm cells appears to be one developmental determinant contributing to seed weight variation between KLS and KSS inbreds and the associated maternal effect on seed weight/size and grain filling in the reciprocal hybrids.

Consistent with the maternal contribution to seed weight/size, transcriptome profiles of reciprocal F_1_ hybrids showed substantial similarities to the maternal parents (Fig. 3). Comparative transcriptional profiling analysis of KSS and KLS inbreds identified a number of DEGs involved in important biological processes. *ZmMRP-1*, one up-regulated gene in KLS inbreds, is so far the only known endosperm transfer cell-specific transcription activator that regulates transfer cell differentiation and associated maternal nutrition allocation (Gómez *et al.*, 2009; Lopato *et al.*, 2014). Thus, the differential expression of *ZmMRP-1* indicated that the divergence of seed size in KLS and KSS might be related to maternally controlled nutrient uptake and allocation during seed development. This also corroborated our hypothesis that maternal sink constraints would set the basis for maternal effect on seed size. Interestingly, GO analysis of DEGs identified that the significantly enriched biological processes in up-regulated DEGs of KLS at 14 DAP were all related to stimulus responses including heat stress. Heat stress imposes limitation on endosperm enlargement, and thus seed size and yield (Folsom *et al.*, 2014). Kernel sink capacity determined by endosperm cell number and/or the number of starch granules is often disrupted by heat stress (Wilhelm *et al.*, 1999; Commuri and Jones, 2001). Thus, the enhanced expression of heat response genes in KLS inbreds may endow the kernels with improved intrinsic ability for thermotolerance, which could contribute to endosperm enlargement and thus more efficient grain filling.

Differential expression and WGCNA analysis both identified DNA methylation as a key process distinguishing large and small seed. We also found a robust association between the DNA methylation GO term and seed size when we compared the current meta-analysis with the previous transcriptional data from Sekhon *et al.* (2014) in which they profiled the transcriptome of the developing endosperm of three large Krug inbreds and three small Krug inbreds. Despite the differences in the exact genetic stocks used and in the tissues sampled between these two studies, we identified largely common GO terms including DNA methylation that were enriched in DEGs between the endosperm of KLS and KSS inbreds at both 15 DAP and 18 DAP (data not shown). DNA methylation is a major epigenetic mark underlying gene imprinting which has been hypothesized to regulate seed size by affecting nutrient uptake and allocation during endosperm development (Costa *et al.*, 2012; Xin *et al.*, 2013; Bai and Settles, 2014). By examining the overlap between DEGs with imprinted genes that were previously identified in developing endosperm (Waters *et al.*, 2013; Xin *et al.*, 2013), we found that a subset of genes differentially expressed at 17 DAP significantly overlapped with a subset of the previously described maternally expressed genes (Supplementary Table S6). Therefore, while our study did not focus on identification of imprinted genes, transcriptional differences in genes controlling DNA methylation provide indirect support for the role of gene imprinting as a molecular mechanism underlying the observed maternal effect on seed size.

Co-expression network analysis also revealed potential biological processes and candidate genes involved in seed development and gene imprinting which could underlie the observed maternal effect on seed size. Co-expression module M12, which was positively correlated with seed weight, contained genes significantly enriched in ovule development. Key genes in M12 included AGAMOUS-LIKE type I MADS-box transcription factor (AGL) genes including *ZAG2*, its paralogous gene *ZMM1*, and the C-type MADS gene *ZMM2* (Fig. 5). *AGL* genes are mostly expressed in female gametophytes or developing seeds, and have been shown to affect endosperm development and regulate seed size (Lu *et al.*, 2012). Studies in Arabidopsis demonstrated that down-regulation of *AGL* genes in the endosperm due to increased levels of homologous siRNAs caused decreased seed size (Lu *et al.*, 2012). Another *AGL* gene, *AGL62,* acting as a dosage-sensitive seed size regulator, correlated positively with seed size (Kradolfer *et al.*, 2013). *ZAG2* of M12 is highly similar to *AGL*5, which was shown to be the direct target of the complex formed by AGAMOUS (AG) and SEPALLATA (SEP) in the control of carpel and ovule development in Arabidopsis. *ZAG2* was also identified as a maternally expressed imprinted gene in maize (Waters *et al.*, 2011; Zhang *et al.*, 2011; Liu *et al.*, 2015) and its expression was largely restricted to developing seeds and endosperm (Supplementary Fig. S3; Sekhon *et al.*, 2011; Stelpflug *et al.*, 2015). Interestingly, module M12 genes were found to be enriched in maternal expressed genes (Supplementary Table S6) by examining the overlap between WGCNA-generated co-expression modules and the imprinted genes identified in developing endosperm (Xin *et al.*, 2013). Considering the similar expression of *ZAG2/ZMM1* between reciprocal hybrids and their maternal plants in addition to previously described imprinted genes contained in module M12, we predict that *ZAG2* is probably a promising candidate gene functioning in regulating seed size through its imprinting role in endosperm. Identifying and separating imprinted loci from reciprocal endosperms is of great interest for future studies and will be greatly beneficial for deciphering the genetic mechanism underlying maternal control of seed size in maize.

Our comprehensive analyses of seed morphology, endosperm cytology, and seed transcriptome revealed a notable role for the maternal parent in determining seed size. The identification of DEGs and co-expression module genes involved in maternal source constraints extends our understanding of the complex molecular and cellular events in this process and provides a foundation for future studies on seed size in crops.

## Supplementary data

Supplementary data are available at *JXB* online.

Figure S1. Gene clustering tree (dendrogram) for identifying consensus modules obtained by hierarchical clustering of adjacency-based dissimilarity based on FPKM values of all RNA-Seq samples.

Figure S2. Heatmaps and barplots of eigengenes for WGCNA-generated co-expression modules (M1, M5, M10, and M13) that were negatively correlated with seed weight.

Figure S3. Spatial and temporal expression of *ZAG2* and *ZMM1* identified by WGCNA-generated co-expression module M12 based on the Maize B73 Gene Atlas.

Table S1. Number of reads, mapping percentage, and number of expressed genes in 24 RNA-seq samples which include four parental inbreds and eight reciprocal F_1_ hybrids collected at 14 DAP and 17 DAP.

Table S2. Gene expression values of B73 RefGen_v2 Filtered Gene Set (FGS) in each sample.

Table S3. Differentially expressed genes and Gene Ontology in KLS and KSS inbred lines.

Table S4. Transcription factors identified in differentially expressed genes between KLS and KSS inbreds.

Table S5. Gene models in co-expression modules significantly associated with seed weight.

Table S6. Relationships of the differentially expressed genes and co-expression modules to the maternally expressed gene sets previously described.

Supplementary Data
