# Supplementary material for: Evidence for maternal control of seed size in maize from phenotypic and transcriptional analysis
Source: J Exp Bot. 2016 Jan 29;67(6):1907–17. doi: 10.1093/jxb/erw006 (PMC4783370; doi:10.1093/jxb/erw006)
Supplement: Supplementary Data [file supp_67_6_1907__index.html]

Evidence for maternal control of seed size in maize from phenotypic and transcriptional analysis — Evidence for maternal control of seed size in maize from phenotypic and transcriptional analysis — Supplementary Data 

# Evidence for maternal control of seed size in maize from phenotypic and transcriptional analysis

## Supplementary Data

Data files

- Supplementary\_table\_S1.xlsx - Supplementary Data
- Supplementary\_table\_S2.xlsx - Supplementary Data
- Supplementary\_table\_S3.xlsx - Supplementary Data
- Supplementary\_table\_S4.xlsx - Supplementary Data
- Supplementary\_table\_S5.xlsx - Supplementary Data
- Supplementary\_table\_S6.xlsx - Supplementary Data
- Supplementary\_figures\_S1\_S3.pdf - Supplementary Data
