# Supplementary material for: Evidence for maternal control of seed size in maize from phenotypic and transcriptional analysis
Source: J Exp Bot. 2016 Jan 29;67(6):1907–17. doi: 10.1093/jxb/erw006 (PMC4783370; doi:10.1093/jxb/erw006)
Supplement: Supplementary Data [file supp_erw006_Supplementary_figures_S1_S3.pdf]

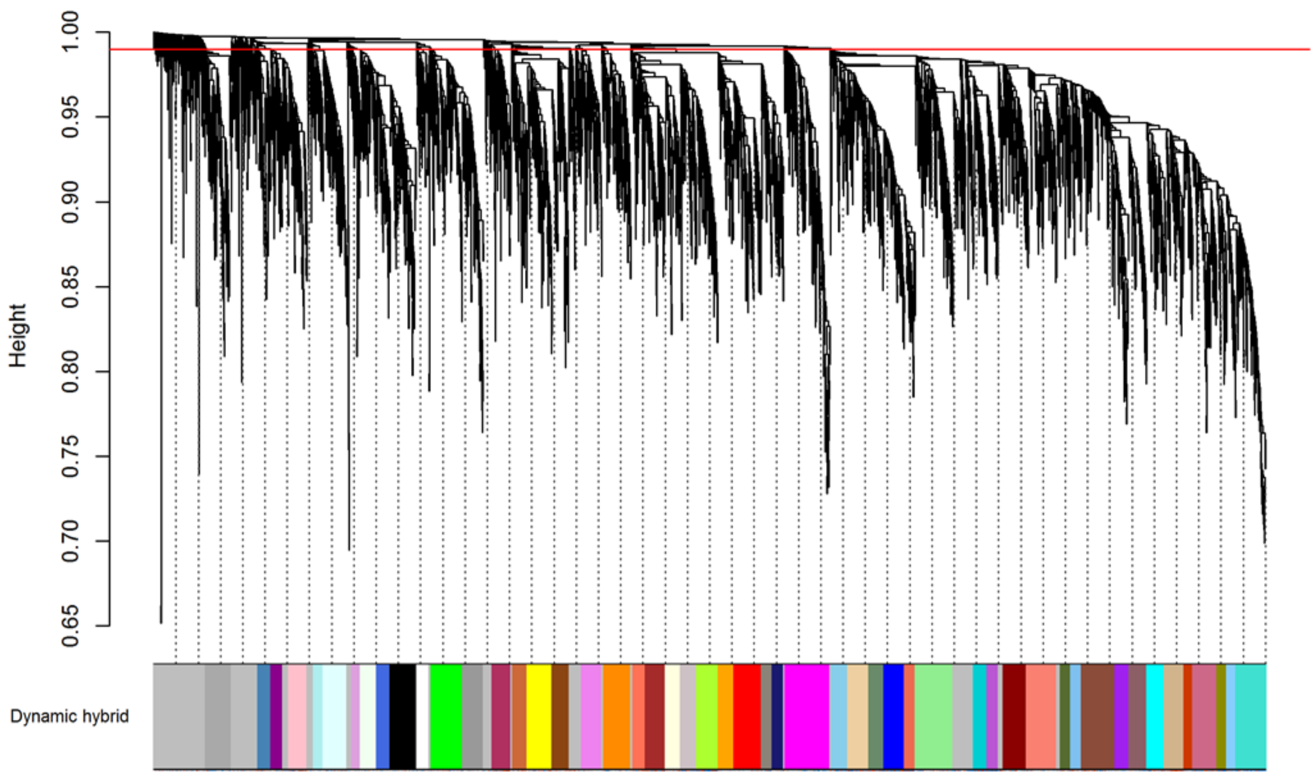

Supplementary Figure S1. Gene clustering tree (dendrogram) for identifying consensus modules obtained by hierarchical clustering of adjacency-based dissimilarity. Branches of the dendrogram, cut at the red line, correspond to consensus modules. Genes in each module are assigned to the same color, shown in the top color band below the dendrogram determined by the Dynamic Tree Cut. Genes not assigned to any of the modules are colored grey.

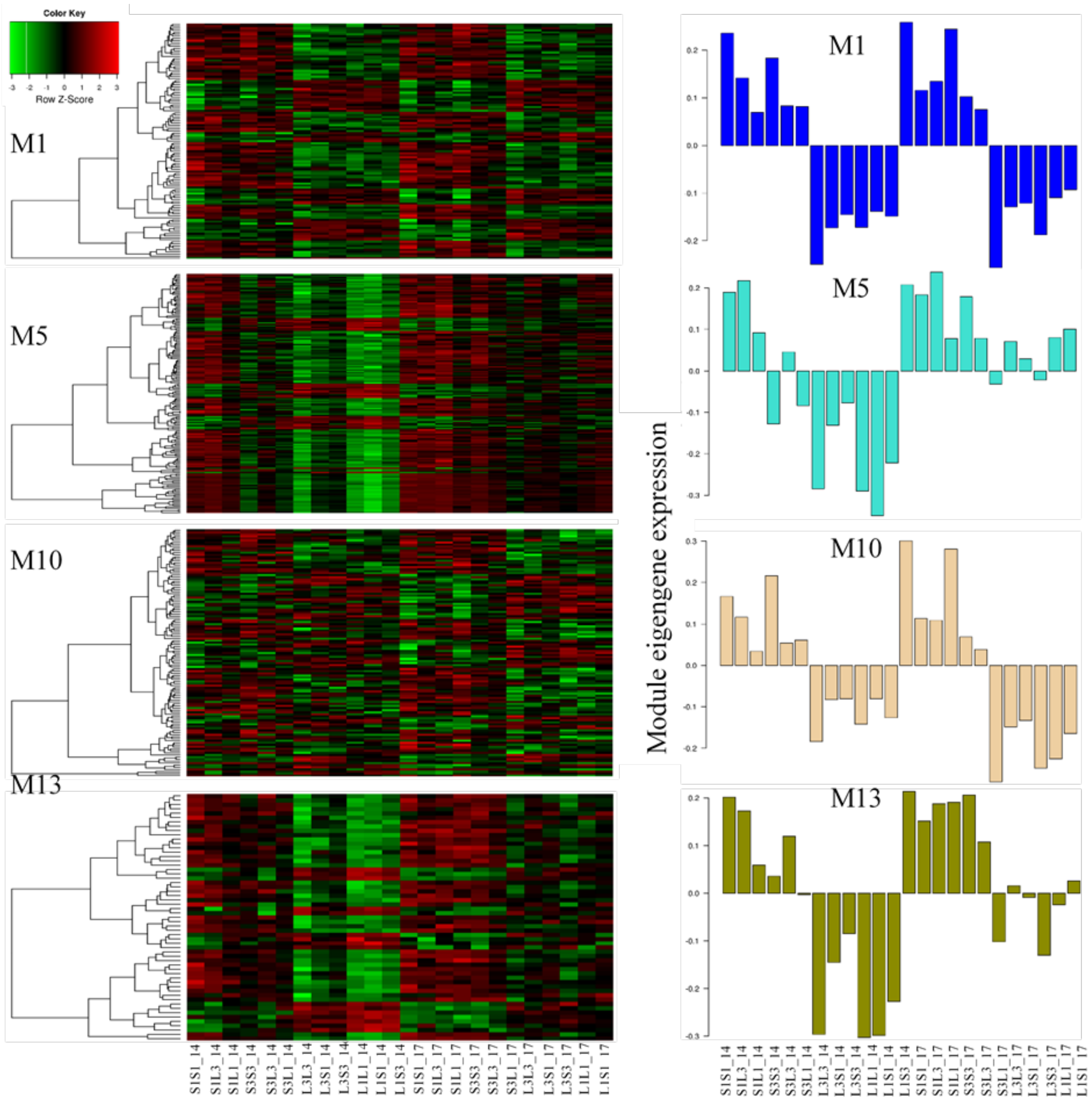

Supplementary Figure S2. Heatmap for representative modules negatively correlated with seed weight and barplot of expression levels of the corresponding eigengene across the samples in the M1, M5, M10, M13. Rows of heatmap (left panel) correspond to genes, columns to samples, red in color key denotes over-expression, green under-expression. _14 and _17 indicate samples collected at 14 and 17 days after pollination (DAP), respectively. S1S1, S3S3, L1L1, and L3L3 are inbred lines derived from KSS30 and KLS30, respectively. F_1 reciprocal hybrids (i.e. S1L1) are designated with the maternal parent on the left and the paternal parent on the right.

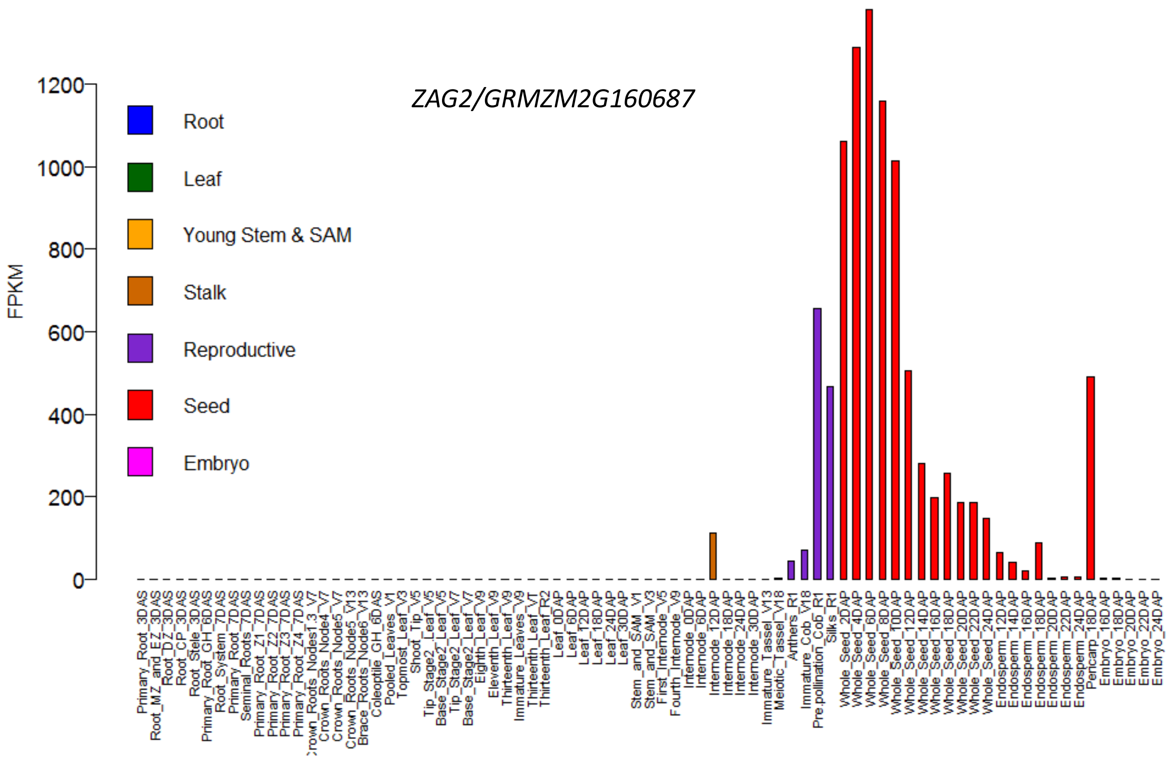

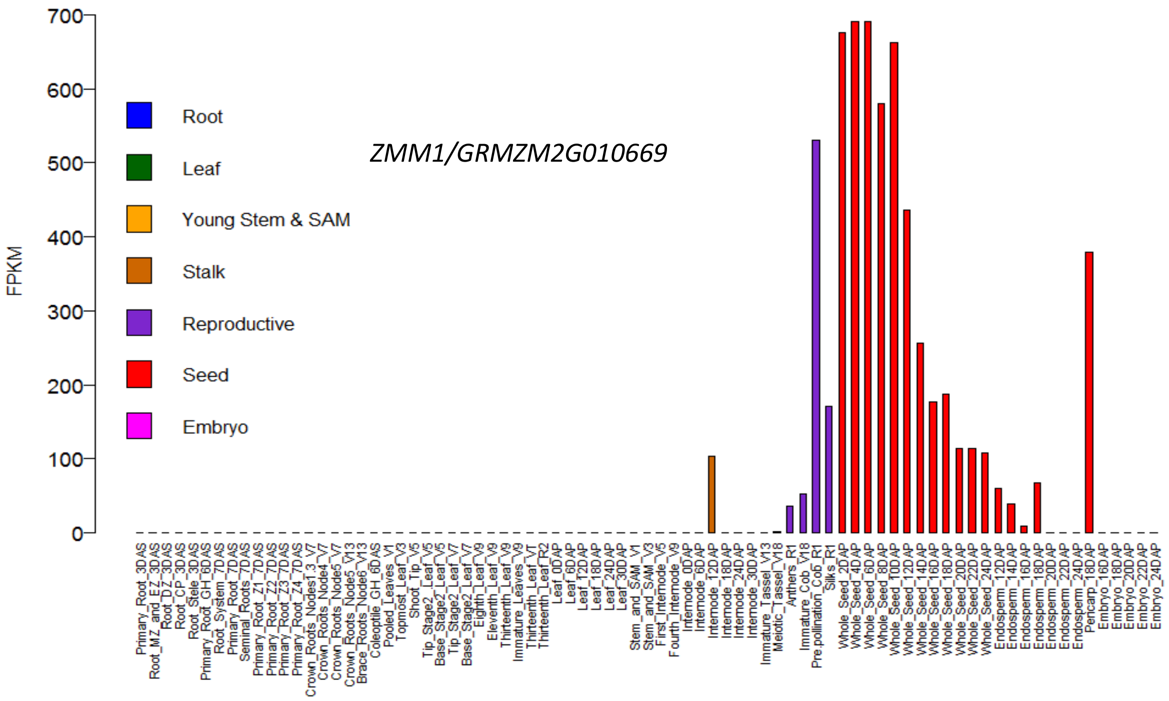

Supplementary Figure S3. Spatial and temporal expression of *ZAG2* and *ZMM1* identified by WGCNA-generated co-expression module (M12) based on the Maize B73 Gene Atlas (Sekon et al., 2011; Stelpflug et al., 2015).
